# Designing Microfluidic-Chip Filtration with Multiple Channel Networks for the Highly Efficient Sorting of Cell Particles

**DOI:** 10.3390/mi15121474

**Published:** 2024-12-05

**Authors:** Myung-Suk Chun

**Affiliations:** 1 Complex Fluids Laboratory, Advanced Materials and Systems Research Division, Korea Institute of Science and Technology (KIST), Seoul 02792, Republic of Korea; mschun@kist.re.kr; Tel.: +82-2-958-5363; 2 Biomedical Engineering Division, KIST School, University of Science and Technology, Seoul 02792, Republic of Korea

**Keywords:** microfluidic-chip, multiple channel, chip design, cell particle sorting, hydrodynamic filtration

## Abstract

Microfluidic-chip based hydrodynamic filtration is one of the passive sorting techniques that can separate cell or particle suspensions into subpopulations of different sizes. As the branch channels and side channels play an important role in maintaining particle focusing, their rational design is necessary for highly efficient sorting. A model framework involving multiple side and multiple branch channels has been developed by extending the analytical analysis of three-dimensional laminar flow in channel networks, which was previously validated by comparison with numerical simulations. Objective parameters were identified as the number of branch channels and each length of individual branches. The presence of multiple side channels causes an increase in the average fluid velocity in main and branch channels as the branch point shifts toward the end of the main channel, which differs from the behavior observed in a single side channel. The number of branches and their individual lengths decrease distinctly in the case of branch channels consisting of narrow and wide sections, which enables the compact design of a microfluidic-chip, being operated by a lower pressure drop under the same throughput. Sorting of bidisperse particles was accomplished with an optimally designed chip to verify this framework by achieving very high recovery and purity.

## 1. Introduction

The sorting of suspended cells or particles has been a subject of significant interest in various biological and clinical researches, biomedical applications, and industrial fine processing. Flow cytometry is a key technology for monitoring the production as well as release of cellular products and clinical use, with accurate and reproducible quality control [1,2]. Although it is constantly improving and growing, conventional flow cytometry is a notoriously variable technique according to clear limitations and troublesome data analysis. For instance, a fluorescent-activated cell sorter (FACS) can offer high purity, but it is bound to be a batch operation, requiring pre-treatments with a subsequent high number of parameters and expensive instruments [3,4,5]. Microfluidic-based sorting techniques of living cells have been developed over the past two decades, which have been proven to have a high throughput through continuous separations without sample damage, inexpensive instruments, faster treatments, and uncomplicated integration with other systems [6,7,8,9,10].

Instead of employing external force fields (e.g., non-uniform electric, magnetic, acoustic forces, etc.) in the active sorting [11,12,13], passive sorting relies on the inherent microfluidic properties underlying the flow field, channel geometry, and particle interactions [14,15,16,17,18,19,20,21,22,23,24]. Furthermore, many investigators have also made attempts to enhance sorting performance by considering either solution characteristics or cell particle properties such as deformability and shape [25,26,27,28,29]. Although passive sorting has the benefit of not using external fields, it is certain that achieving satisfactory efficiency would be a breakthrough in passive sorting. Improved sorting efficiency is actually related to the channel design, allowing design protocols regarding channel networks, flow field, and fluid properties.

**Figure 1:**
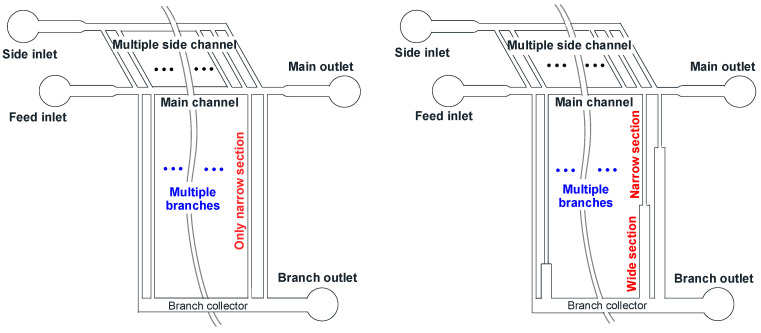
HDF microfluidic-chip for cell particle sorting with layout of multiple sides and multiple branches, where each branch can be designed with only narrow section (**left**) as well as consisting of narrow and wide sections (**right**).

As one of the passive sorting techniques, hydrodynamic filtration (HDF) has been shown to effectively either separate or isolate rigid particles and deformable living cells by differences in size [14,18,19,21,22]. The mechanism of HDF is comparable to conventional crossflow filtration such as nanofiltration, ultrafiltration, and microfiltration. Here, suspended cell particles are continuously introduced into the main channel and well-designed branch channels perpendicular or tangential to the main channel remove the feed suspension. Branch channels function similarly to membrane pores, whether they are arranged in an ordered or disordered type. The multiple branches play a role in particle focusing closer to the sidewalls of the main channel, ensuring that only the stream near the sidewall enters the branches. Considering the additional contribution of the side channel in particle focusing, the selective extraction of streamlines caused by side channel flow is controlled by the flow fraction at each branch for sorting. It is pointed out that a single side channel has been designed in all previous studies. In such cases, the flow focusing tends to weaken towards the end of the main channel, providing a reduction in sorting efficiency. This behavior can also occur when a viscoelastic fluid is introduced. From the design viewpoint to solve this problem, the flow focusing can be maintained at all points of the main channel by introducing multiple side channels, as displayed in Figure 1.

In this study, the rational design of an HDF chip is presented for efficient sorting by exploiting the three-dimensional (3D) analytical model regarding microfluidic networks of rectangular channels consisting of multiple sides and multiple branches. In order to prevent unwanted reverse flow during particle discharge, a wide section is incorporated into the branch channel, designed with a relatively larger width to act as the reservoir. The advantage of the wide section was examined by comparing cases where the wide section is present and absent. The effect of branch structure on the chip design is quantified by estimating the flow velocity, the number of branch channels, and the length of each individual branches. The proposed model framework achieves an improvement, where volumetric flow rate (i.e., average velocity) and pressure drop at each branch point can sequentially be estimated for arbitrary conditions, thereby eliminating the need for the complicated iterative steps required in earlier work [18]. In order to perform experimental verifications, bidisperse spherical particles were sorted by employing an HDF chip designed with the proposed model framework. Sorting efficiency was quantified with the recovery of different outlets for each target particle and the purity of each target particle at these outlets. Possible applications in biomedical and industrial contexts are also discussed.

## 2. Theoretical Model

**Figure 2:**
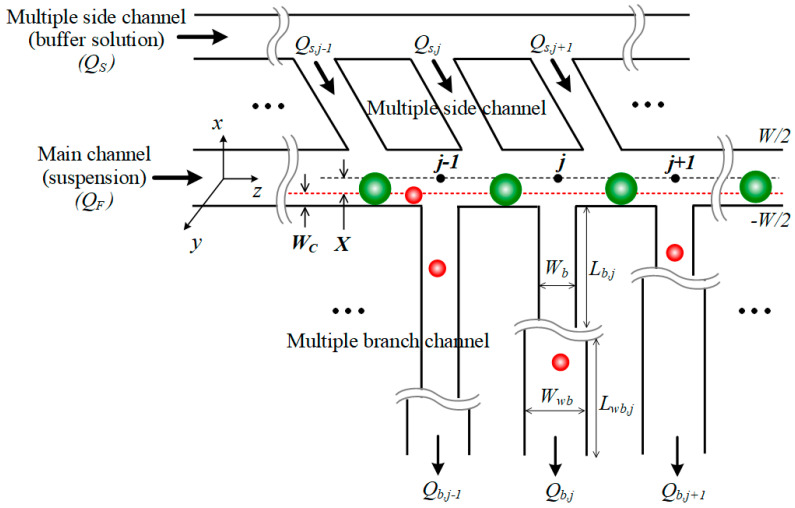
Top-view schematic of the channel network design for bidisperse or bimodal sorting. Small cell particles (red) flow into the branch channel, while large cell particles (green) pass along the main channel. A coordinates system incorporates the branch point index j, and the number of branch channels *N_b_* is equal to the number of side channels.

From the fluid mechanics perspective, pressure-driven flow channel networks can be modeled with an analytical approach in terms of hydraulic flow resistance, analogous to the electric circuit theory [30,31]. The cell particle suspension is fed into the main inlet with flow rate *Q_F_*, while the simple fluid of the buffer solution is supplied into the side inlet with *Q_S_*. As the flow fraction determines the gradual removal of the stream from the main channel, sorted cell particles through the multiple branches are collected into the branch outlet. The remaining particles in the main channel are collected into the main outlet. In Figure 2, an abrupt condition is taken that the number of side channels is equal to the number of branch channels *N_b_*.

### 2.1. Flow Fraction in a Rectangular Channel for Hydrodynamic Filtration

The present model framework starts a fully developed flow applied along a main straight channel with width *W*, height *H*, and channel length *L*, which is much longer than the entrance length (i.e., 0.1*r_h_*Re) [21]. Here, Re is the Reynolds number and the equivalent hydraulic radius *r_h_* (=*WH*/(*W* + *H*)) is larger than the cell particle radius *a_p_*. As described in the previous study [18], the inertial lift force of a suspended particle [32,33] is negligible, since the particle Reynolds number Re_p_ (=Re(*a_p_*/4*r_h_*^2^)) becomes less than unity. Mixing of the feed and side streams is quite slow due to stronger convection compared to diffusion, according to the higher Péclet number of order 10^3^ [34,35]. All notations used here are detailed in Appendix A as the nomenclatures.

For the steady-state Newtonian fluid flow with viscosity *µ*, the velocity field **u** driven by pressure gradient ∇*P* (=Δ*P*/*L*) is governed by Stokes’s equation: *μ*∇^2^**u** = ∇*P*. The velocity profile through a rectangular channel with no-slip boundary condition can be expressed as a Fourier series form [30].

(1) $$u_{z}\left(x,y\right)=\frac{4H^{2}}{\pi^{3}\mu }\frac{\Delta P}{L}\sum_{n=odd}^{\infty }\frac{1}{n^{3}}\left[1-\frac{\cosh(n\pi x/H)}{\cosh(n\pi W/2H)}\right]\sin\left(n\pi y/H\right).$$

Integrating Equation (1) over −*W*/2 ≤ *x* ≤ *W*/2 and 0 ≤ *y* ≤ *H* becomes the volumetric flow rate *Q* through a channel cross-sectional area as *Q* = (*WH*^3^/12*μ*)(∆*P*/*L*)*f*(*H,W*). Here, ∆*P*/*Q* corresponds to the hydraulic resistance and the flow function *f* is given by

(2) $$f=1-\frac{192}{\pi^{5}}\frac{H}{W}\sum_{n=odd}^{\infty }\frac{1}{n^{5}}\tanh\left(n\pi W/2H\right).$$

In Figure 2, a cut-off width, *W_C_*, is set as the virtual boundary of the fluid layer that will divide the total streams into two parts. Then, one can derive the fractional flow function *f*_f_ by integrating over a partial fraction of −(*W*/2 − *W_C_*) ≤ *x* ≤ *W*/2 − *W_C_* and 0 ≤ *y* ≤ *H* and denoting *X* = *W*/2 – *W_C_*.

(3) $$f_{f}=\frac{2X}{W}-\frac{192}{\pi^{5}}\frac{H}{W}\sum_{n=odd}^{\infty }\frac{1}{n^{5}}\frac{\sinh\left(n\pi X/H\right)}{\cosh\left(n\pi W/2H\right)}.$$

As a result, the channel networks can be analyzed along with the analogy for the volumetric flow rate.

### 2.2. Microfluidic-Chip Designing with Multiple Channel Networks

As a single branch channel hardly attains high-efficiency sorting due to insufficient particle focusing, the design of multiple branches with the branch point *j* is crucial for aligning and concentrating the particles by fluid removal along the main channel. In Figure 2, note that the total input flow is the sum of *Q_F_* and *Q_S_* (=*Q*_s,*j*_*N_b_*). The channel height *H* remains uniform throughout the entire channel network, and equally spaced branch channels share the same width, *W_b_*, that is smaller than the main channel width *W*. The flow at a reference point (*j* = 0) is assumed to start forming a fully developed parabolic profile, justified by Equation (1). This assumption is based on the fact that the distance from the junction of the main and side channels to the first branch point is designed to be larger than the entrance length.

The flow stream diverted into the first branch channel *Q_b_*_,1_ at *j* = 1 is identified as the partial volumetric flow rate for the focused stream in the range −*W*/2 ≤ *x* ≤ −(*W*/2 − *W_C_*). Invoking that the flow stream remained after the branch point is toward a following main channel, the fractional flow function in the branch *f*_b_ (=(*f* − *f*_f_)/2) can be derived from Equations (2) and (3),

(4) $$f_{b}=\left(\frac{W-2X}{2W}\right)-\frac{192}{\pi^{5}}\frac{H}{W}\sum_{n=odd}^{\infty }\frac{1}{n^{5}}\frac{\sinh\left(n\pi (W-2X)/4H\right)\cosh\left(n\pi (W+2X)/4H\right)}{\cosh\left(n\pi W/2H\right)}.$$

Here, the ratio of the flow fraction *ξ* is defined as the ratio of the flow rate between the branch stream and main stream. *ξ* at *j* = 1 can be expressed as *Q_b,_*_1_/(*Q*_m,0_ + *Q_s,_*_1_) with the frontal flow rate in the main channel (=*f*_b_/*f*). *f*_b_ becomes zero if the flow streams in the main channel are not diverted (i.e., *W_C_* = 0), whereas *f*_b_ becomes *f* if all the streams in the main channel flow into the branch channel (i.e., *W_C_* = *W*). Recalling Equation (4) in the first branch, the branch flow and the remaining flow can be applied for entire channel networks,

*Q_b,j_* = *ξ*(*Q_m,j_*_−1_ + *Q_s,j_*), (5)

*Q_m,j_* = (1 − *ξ*)(*Q_m,j_*_−1_ + *Q_s,j_*). (6)

All branch points take a constant *ξ*, owing to the consistent cut-off width regardless of different flow rates at each point.

The pressure drops between *j* = 0 and each outlet is equivalent on the implication that the main and all branch channels are open to the atmosphere, yielding

(7) $$\sum_{j=0}^{k-1}\Delta P_{m,j}+\Delta P_{b,k}=\sum_{j=0}^{N_{b}}\Delta P_{m,j}=\Delta P\;\;for\;\;k=1∼N_{b}.$$

Δ*P_m,j_* indicates the pressure drop from the (*j* − 1)th to the *j*th branch point and Δ*P_b,k_* indicates the pressure drop from the *k*th branch point to the branch outlet. From the ratio of flow fraction *ξ* at the *j*th branch point, unknown values of *Q_b,j_* and *Q_m,j_*_+1_ can be subsequently computed by applying *Q_m,j_*_+1_ = (1 − *ξ*)(*Q_m,j_* + *Q_s,j_*_+1_). A pressure drop along the main channel is estimated from

(8) $$\Delta P_{m,j}=\left(12\mu /WH^{3}\right)\left[Q_{m,j}L_{m,j}/f\left(H,W\right)\right],$$

providing a total pressure drop in channel networks Δ*P*. Combining the pressure drops at each branch by Equation (7), each length of individual branch channels can be obtained from the relationship between *Q_b,j_* and flow function *f*(*H*,*W_b_*), expressed by

(9) $$L_{b,j}=\frac{W_{b}H^{3}}{12\mu }\frac{\Delta P_{b,j}}{Q_{b,j}}\left[1-\frac{192}{\pi^{5}}\frac{H}{W_{b}}\sum_{n=odd}^{\infty }\frac{1}{n^{5}}\tanh\left(\frac{n\pi W_{b}}{2H}\right)\right].$$

Now, let us consider the branch channel designed to have a narrow section and wide section, with a narrow section width *W_b_*, narrow section length *L_b_*, wide section width *W_wb_*, and wide section length *L_wb_*. The narrow section exists for the actual application of a pressure drop and the wide section works as the reservoir. The total lengths of each channel are uniform by adjusting their hydraulic flow resistances. Then, the flow stream diverted into each branch channel is explicitly given by identifying the relationship between flow resistance and flow function,

(10) $$Q_{b,j}=\left(\frac{W_{b}H^{3}}{12\mu }\frac{\Delta P_{b,j}}{L_{b,j}}\right)f\left(W_{b}\right)=\left(\frac{W_{wb}H^{3}}{12\mu }\frac{\Delta P_{b,j}^{wb}}{L_{wb,j}}\right)f\left(W_{wb}\right).$$

Here, the first branch channel (i.e., *j* = 1) is set to be formed only with a narrow section. Then, the lengths of narrow and wide sections for the first branch channel can be derived,

(11) $$L_{b,1}=\frac{W_{b}H^{3}}{12\mu }\frac{\Delta P_{b,1}}{Q_{b,1}}f\left(W_{b}\right),$$

(12) $$L_{wb,1}=0.$$

In consequence, the lengths of each section for individual branch channels (i.e., *j* = 2~*N_b_*) can be derived, respectively.

(13) $$L_{b,j}=\left(L_{b,1}-\frac{W_{wb}H^{3}}{12\mu }\frac{\Delta P_{b,j}}{Q_{b,j}}f\left(W_{wb}\right)\right)/\left(1-\frac{W_{wb}f(W_{wb})}{W_{b}f(W_{b})}\right),$$

(14) $$L_{wb,j}=L_{b,1}-L_{b,j}$$

Note that the constant fluid viscosity is considered in the present model. However, cell particle suspensions are often more viscous than the simple fluid of buffer solution such that the fluids supplied into the feed inlet and side inlet are likely to develop a two-phase Newtonian fluid or generalized Newtonian fluid with rheological properties [36]. To explore the effect of two-phase flow, further derivations are necessary as a function of viscosity ratios between the feed and side flows.

### 2.3. Computation Scheme

**Figure 3:**
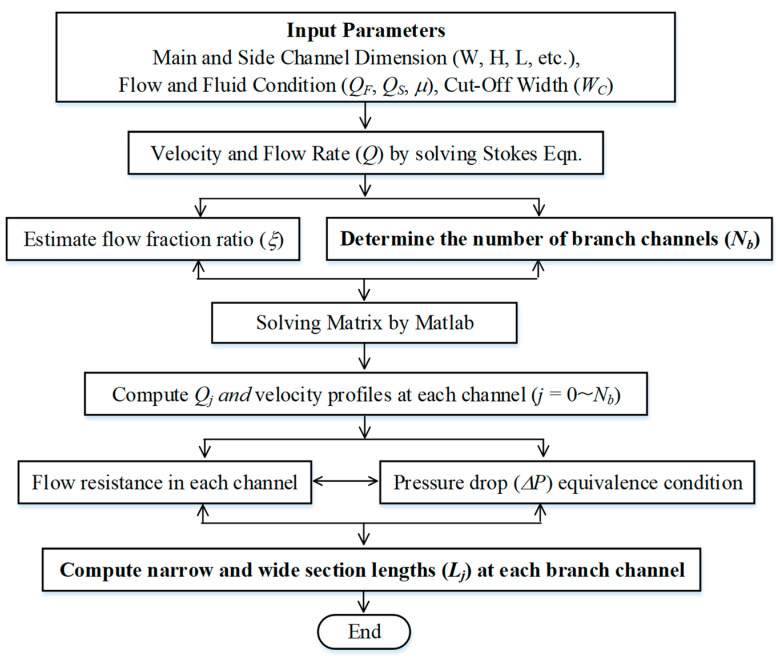
The computation algorithm of the present model framework.

Figure 3 presents the overall algorithm employed in this study. Objective parameters for optimum designing are the number of branch channels and each length of individual branch channels. The number of branches can be determined based on a criterion involving a constant residence time of suspended cell particles traveling in the channel network. The residence time is represented by the longest traveling time through the last branch with the lowest flow velocity, under the same throughput. Detailed information can be found in the previous study [21]. While increasing the number of branches improves the selectivity of cell particles, it undesirably results in a larger chip size.

Table 1 summarizes the input parameters and their values applied in this study. Illustrative computations are performed, providing a side-flow-to-main-flow ratio (*Q_S_*/*Q_F_*) of above 3, which can be a favorable operation condition. The cut-off width *W_C_* is set to half of the target cell particle size, expecting that their hydrodynamic center less than *W_C_* would be removed through the multiple branches connected to the branch outlet. Instead of invariant *W_C_*, *W_C_* can be adjusted to varying values at each branch point depending on the particle size distribution, such as tridisperse, tetradisperse, and polydisperse distributions. This analytical model framework was solved with the implementation of Matlab R2024b (Mathworks, Natick, MA, USA). As was performed in the previous study [21], design properties obtained from this framework can be validated against numerical results obtained by flow simulations. 

## 3. Materials and Methods

After determining all values of the channel dimension, the photomask was prepared, and then the HDF chip was fabricated by employing standard soft lithography and oxygen plasma bonding. SU-8 2150 photoresist (Microchem, Newton, MA, USA) and SU-8 developer (Microchem, Newton, MA, USA) were used for the master mold prepared in the KIST Micro-Nano Fabrication Center. A polydimethylsiloxane (PDMS) base and curing agent (Sylgard 184 silicone elastomer kit, Dow Corning, Midland, MI, USA) were mixed at a 10:1 ratio for the channel replica, which was well bonded to a slide glass using an oxygen plasma generator (CUTE-1 MP, FemtoScience, Hwaseong-si, Republic of Korea).

According to the present design with a *W_C_* of 33 μm, the dispersed spherical particles are designed to be separated into small particles with a diameter less than 66 μm and large particles with a diameter above 66 μm. The model suspension was prepared by fluorescent polystyrene latex beads (Thermo Fisher Scientific Inc., Waltham, MA, USA) of 25 μm (red) and 70 μm (green) in diameter dispersed in a surfactant solution of 0.2% (*w*/*v*) Triton X-100. These rigid beads are highly monodispersed (cf., polydispersity < 1.05) and those diameters correspond to the normal size of cells [29,31]. In the case of the cell sorting, the buffer solution should be applied instead of surfactant solution.

This bidisperse particle suspension with equivalent-number concentrations was diluted as 5 ppm and then introduced into the feed inlet using a syringe pump (Pump 11 Elite–Nanomite, Harvard Apparatus, Holliston, MA, USA), while the surfactant solution was injected into the side inlet using another syringe pump (Fusion 200, Stafford, TX, USA). A hemocytometer and cover slide were prepared for counting the number of particles collected from each outlet, in which the sample volume was also measured. The feed and sorted particle solutions were visualized by an inverted fluorescence microscope (Eclipse TE2000-U, Nikon, Tokyo, Japan) with a 10× objective lens (numerical aperture of 0.3). All data were obtained through at least three independent experiments, and ImageJ 1.53v software (NIH, Bethesda, MD, USA) was used to analyze the sorting data.

## 4. Results

### 4.1. Computation-Design Results

**Figure 4:**
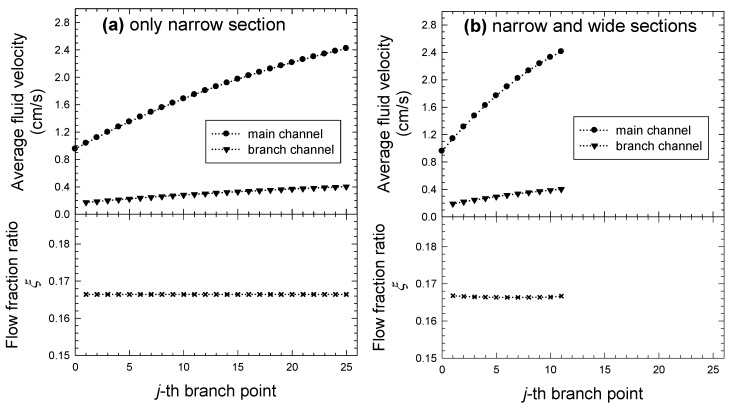
The variations in average velocity at each branch point for main and branch channels and corresponding estimated flow fraction ratio, where each branch channel is designed with (**a**) only narrow section and (**b**) consisting of narrow and wide sections.

Two cases are considered as the branch channels formed by only a narrow section as well as a branch channel formed by wide and narrow sections. Figure 4 illustrates the average velocity in the main and branch channels, computed at each branch point, along with the flow fraction ratio estimated from these values. The number of branch channels is obtained as 25 for the case with only a narrow section (Figure 4a), and 11 for the case with narrow and wide sections (Figure 4b). The average velocity increases in the main and branch channels as the branch point becomes larger (i.e., towards the downstream position of the main channel), regardless of the presence of a wide section. This behavior is evidently different from the case of a single side channel. The velocity increase is due to the continuous replenishment of fluids from multiple side channels having the same number of branch channels, as it goes to the downstream position of the main channel. The degree of velocity increase is about 260% in the main channel and about 200% in the branch channel, indicating a greater increase in the main channel. These results imply that the depletion of main flow never occurs, even at the end of the main channel, which is also confirmed by the result of a constant flow fraction ratio. Furthermore, it means that the cut-off width assigned in the main channel can be maintained consistently from the start to the end of the main channel.

**Figure 5:**
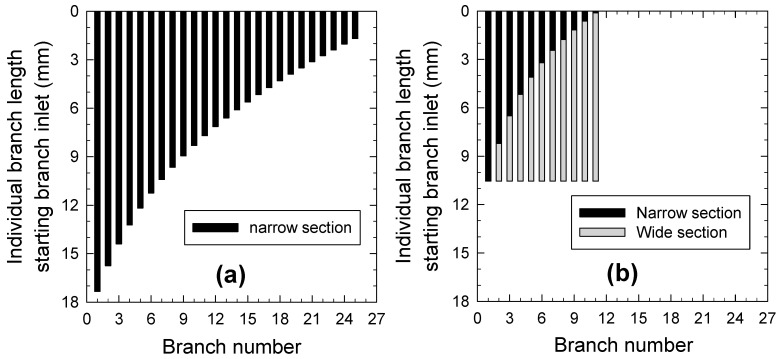
Computational results regarding variations in channel lengths at each branch point j for multiple branches designed with (**a**) only narrow section and (**b**) consisting of narrow and wide sections.

Figure 5a and Figure 5b show the computed lengths of each branch channel for the two cases described above. In the case with only a narrow section, the first branch channel has a maximum length of 17.4 mm and the branch channel becomes monotonically shorter as it goes downstream. In comparison, in the case with both narrow and wide sections, the length of the narrow section decreases toward the downstream branch channels, while the length of the wide section increases accordingly, resulting in a constant total length of 10.5 mm for all branch channels. It should be noted that applying a wide section in the branch channel offers the advantage of sorting with a simplified and compact channel design, characterized by fewer and shorter branch channels.

### 4.2. Sorting System and Efficiency

**Figure 6:**
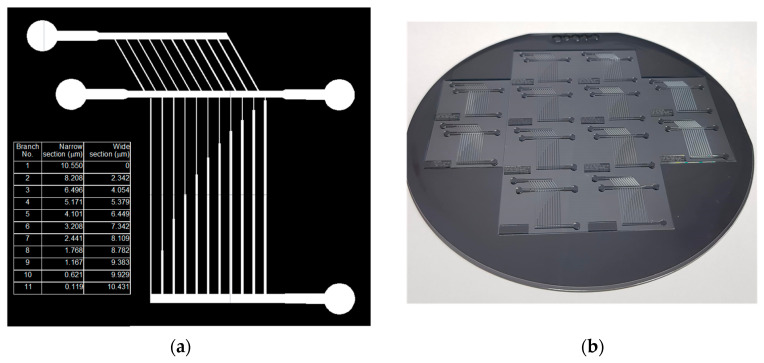
(**a**) A layout of photomask for fabricating a microfluidic-chip designed with multiple side and multiple branch channels consisting of narrow and wide sections to sort out bidisperse particles, where detailed dimension values are given in Table 1; (**b**) picture of master mold fabricated by MEMS process.

As an experimental verification of the present model framework, a sorting experiment was performed with a HDF chip fabricated with the computed design values. The design was accurately drawn by a computer-aided program (AutoCAD 2019), and a film mask supplied by the photomask manufacturer was prepared, as displayed in Figure 6a. Values were provided for the lengths of the narrow section and wide section at each branch. After soft lithography with this mask, a master mold containing negatively patterned photoresist can be obtained, as depicted in Figure 6b.

**Figure 7:**
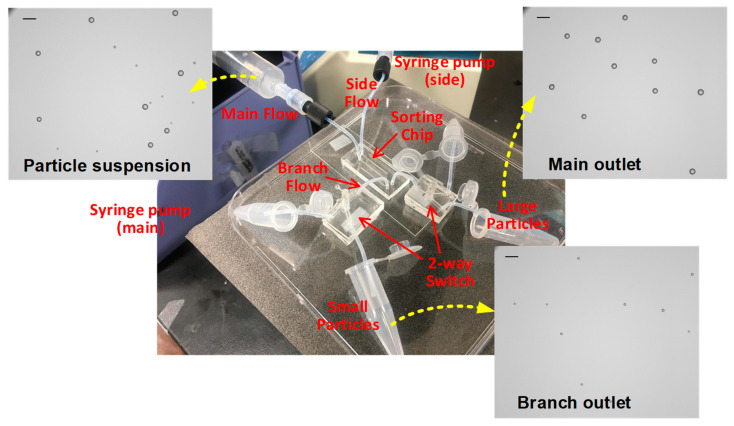
The experimental setup with microfluidic-chip filtration device and micrographs of sorting results of bidisperse particle suspensions before separation and after collection from each outlet, where scale bar is 380 μm.

The experimental setup for the prototype of our sorting is demonstrated in Figure 7. Switching valves were adopted to operate conveniently in either separation mode or collection mode. Separated particles were collected from each outlet, and their numbers could be counted by analyzing the images. Figure 7 shows the microscopic views of particles before separation and recovered from each outlet, where small and large particles were collected from the branch outlet and main outlet, respectively.

**Figure 8:**
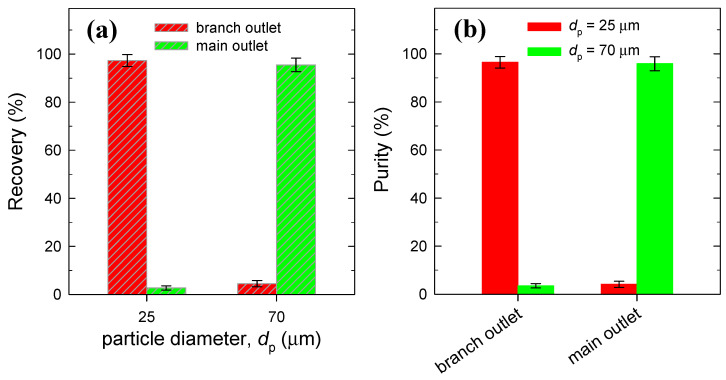
Sorting results with (**a**) the recovery distribution of particles according to the outlet and (**b**) the purity at each outlet. Each value represents the mean ± SE from three experiments.

As is presented in Figure 8a and Figure 8b, the recovery and purity can be estimated by counting the number of particles collected from each outlet with the sample volume, accessing sorting efficiency or performance. Samples from each outlet were loaded onto a hemocytometer by using a pipette, where a cover slide was placed to ensure uniform distribution of the suspension, and then microscope images of particles within the defined counting grids were captured. Using the ImageJ software mentioned above, particles falling within a size range were categorized and counted accordingly. The recovery is defined as the ratio of the number of target particles collected from a particular outlet to the total number of target particles in the sample collected from all outlets, providing information as to which outlet a particular particle is collected from. The purity is the ratio of the number of target particles to the total particle number in the collected sample at each outlet, representing which particles are more prevalent at each outlet. If the particle radius is larger than the cut-off width of 33 μm, it flows through a main channel. On the basis of this condition, small (25 μm) and large (70 μm) particles were collected almost always from the branch outlet and main outlet with a recovery of ca. 97% and 95%, respectively. Populations of small and large particles were collected in the purity of ca. 96% and 96% from the branch outlet and main outlet, respectively.

To examine the validity of the proposed channel design, sorting was performed with the well-known model particle of latex beads in this study. This framework provides ultimately useful information on HDF chip design for precise and efficient cell sorting. Hence, we will conduct sorting experiments by applying deformable cells, enabling broader applications in biomedical and industrial contexts, which should be a further study. For instance, the effective separation of pancreatic islets from acinar cells holds significant potential for diabetes treatments, while the accurate fractionation of specific stem cell subpopulations could advance regenerative medicine and cell-based therapies. In addition, the ability to reliably sort cells in a compact and low-pressure system highlights the potential for scalable manufacturing processes in the biotechnology and pharmaceutical industries.

## 5. Conclusions

An extended model framework for HDF was developed with the aim of improving sorting efficiency, building on a previous analytical analysis of the 3D laminar flow field in complicated channel networks. The present model framework conducts more complicated system involving multiple sides and multiple branch channels. By solving with the sequential scheme, the number of branch channels and each length of the narrow and wide sections of individual branches were determined as objective parameters for optimum design.

According to the analytical model for flow fields and practical designing, we estimated an average velocity and the ratio of flow fraction at each branch point. Unlike the case of a single side channel, the effect of multiple side channels allows that the average fluid velocity increases in the main and branch channels as the branch point approaches the end of the main channel. It should be emphasized that the branch channel consisting of narrow and wide sections leads to the design of a smaller-sized microfluidic-chip being operated by a lower pressure drop, owing to the reduced number of branches as well as their lengths. A preliminary experiment for sorting bidisperse particles was performed using an optimally designed chip, resulting in the proposed framework being validated by achieving very high purity and recovery.

## Figures and Tables

**Table 1:** Input parameters and their values for computation-design framework.

Parameters	Main Channel	Side Channel	Branch Channel	
			Narrow Section	Wide Section
*H* (μm)	350	350	350	350
*W* (μm)	400	100	80	160
Inter-channel distance (μm)	-	500	540	460
Total length (mm)	14.5	3	10.55	
Slanted angle (degree)	-	60	90	
*W_C_* (μm)	33	-	-	
*Q* (μL/min)	<80	<250	-	

## Data Availability

The data that support the findings on this study are available from the corresponding author upon reasonable request.

## Appendix A

As the nomenclatures, the followings are used in this manuscript:

*a_p_*	radius of particle [μm]
*f*	flow function [-]
*f*_b_	fractional flow function in branch channel [-]
*f*_f_	fractional flow function [-]
*H*	channel height [μm]
*j*, *k*	branch point along the main channel [-]
*L*	total length of main channel [mm]
*L_b,j_*	narrow-section length of *j*th branch channel [mm]
*L_m,j_*	main-channel length from (*j* − 1)th to *j*th branch point [mm]
*L_wb,j_*	wide-section length of *j*th branch channel [mm]
*N_b_*	number of branch channels [-]
*n*	coefficient for Fourier series form [-]
Δ*P*	total pressure drop [Pa]
Δ*P_b,j_*	pressure drop at *j*th branch channel [Pa]
Δ*P_b,k_*	pressure drop in narrow section from *k*th branch point to branch outlet [Pa]
Δ*P^wb^_b,k_*	pressure drop in wide section from *k*th branch point to branch outlet [Pa]
Δ*P_m,j_*	pressure drop from (*j* − 1)th to *j*th branch point [Pa]
*Q*	volumetric flow rate [μL/min]
*Q_b,j_*	volumetric flow rate at *j*th branch channel [μL/min]
*Q_F_*	volumetric flow rate of feed [μL/min]
*Q_m,j_*	volumetric flow rate at main channel in front of *j*th branch point [μL/min]
*Q_S_*	volumetric flow rate of side inlet [μL/min]
*Q_s,j_*	volumetric flow rate at side channel in front of *j*th branch point [μL/min]
*r_h_*	equivalent hydraulic radius [μm]
**u**	fluid velocity vector [mm/s]
*W*	main-channel width [μm]
*W_b_*	narrow-section width of branch channel [μm]
*W_C_*	cut-off width [μm]
*W_wb_*	wide-section width of branch channel [μm]
*X*	*W*/2 − *W_C_* [μm]
*x*	spanwise (lateral) direction [μm]
*y*	longitudinal direction [μm]
*z*	streamwise (axial) direction [mm]
Greek symbols	
*μ*	fluid viscosity [Pa·s]
*ξ*	flow fraction ratio [-]
